# Detection of carbapenem resistance mechanisms in difficult-to-treat *Pseudomonas aeruginosa* clinical isolates from a tertiary care hospital in Egypt

**DOI:** 10.1186/s12866-026-05550-2

**Published:** 2026-09-04

**Authors:** Dalia Salem, Manar Zaiton, Ahmed El-Shenawy, Heba Dahroug, Samah El-Dein, Doaa Gamal, Azza Ali Althoqapy, Amr AW. Mohamed, Mohamed A. Shemis, Manal Diab, Soheir S. Maklad

**Affiliations:** 1 Microbiology Department, Theodor Bilharz Research Institute (TBRI), P.O box 12411, 30 Imbaba, Giza, Egypt; 2 Medical Microbiology and Immunology, Faculty of Medicine for Girls, Al- Azhar University, Cairo, Egypt; 3 Biochemistry and Molecular Biology Department, Theodor Bilharz Research institute (TBRI), P.O box 12411, 30 Imbaba, Giza, Egypt

**Keywords:** Carbapenem resistant *Pseudomonas aeruginosa* (CRPA), Difficult-to-treat resistance (DTR), Multidrug resistance (MDR), Carbapenemases, *bla*_NDM_, Efflux pumps, *MexB*, *MexY*, *OprD*.

## Abstract

**Background:**

The increasing incidence of carbapenem-resistant *Pseudomonas aeruginosa* (CRPA) poses a significant global health concern, particularly with the emergence of difficult-to-treat resistance (DTR), which seriously restricts therapeutic options.

Therefore, we aimed to characterize DTR-PA isolates among the studied CRPA; regarding their resistance mechanisms to carbapenems and their antimicrobial susceptibility profiles.

**Methods:**

Fifty-two non-duplicate CRPA isolates were collected from clinical specimens. Antimicrobial susceptibility testing was performed using disc diffusion and VITEK2 compact system. Isolates were classified as multidrug-resistant *P. aeruginosa* (MDR-PA), extensively drug-resistant *P. aeruginosa* (XDR-PA), and DTR-PA. Conventional PCR was used to detect carbapenemase genes (*bla*_NDM_, *bla*_VIM_, *bla*_IMP_, *bla*_KPC_, and *bla*_OXA-48_). Quantitative real-time PCR (qRT-PCR) identified the over expression of efflux pump genes (*mexB*,* mexY*) and the down regulation of outer membrane porin (*oprD*) gene.

**Results:**

Fifty-two (52) CRPA isolates were categorized according to resistance phenotype into three main groups where 46% were identified as DTR-PA, 38% as XDR-PA, 8% as MDR-PA and 8% remained uncategorized. In DTR-PA, carbapenemase genes were detected in 92% of isolates with *bla*_*NDM*_ being the most prevalent (75%), followed by *bla*_VIM_ (54%) and *bla*_OXA-48_ (38%). Co-existence of multiple carbapenemase genes was common. Efflux pump mechanism was the most common mechanism of resistance as overexpression of *MexB* and *MexY* was observed in 98% and 71% of isolates, respectively, whereas downregulation of *oprD* was detected in 92%. The DTR-PA phenotype demonstrated the least sensitive profiles and most of the isolates (92%) were positive to all the studied resistant mechanisms (carbapenemase production, overexpression of efflux pump, and porin loss) with a statistically significant association (*P* = 0.001).

**Conclusions:**

Difficult to treat *Pseudomonas aeruginosa* is highly prevalent among CRPA isolates in our setting and is caused by multiple resistance mechanisms, particularly *bla*_NDM_ carriage, efflux pump overexpression, and *oprD* downregulation. These findings underscore the urgent need for enhanced molecular surveillance and antimicrobial stewardship to limit further dissemination of highly resistant *Pseudomonas aeruginosa* (*P. aeruginosa)* iaolates. Future studies utilizing the DTR-PA definition are needed, as this category highlights the most resistant CRPA isolates with maximum resistance profiles that most likely to pose clinical challenges.

## Background

*Pseudomonas aeruginosa* (*P. aeruginosa*) is a Gram negative non fermenting bacterial rod and a common opportunistic pathogen responsible for causing hospital mortality ranges from 10.5% to 29.6% [1]. The rapid emergence of carbapenem resistant *Pseudomonas aeruginosa* represents a major global public health concern, as carbapenems are frequently kept as last-resort treatments for multidrug-resistant (MDR) Gram-negative infections [2]. Carbapenem antibiotics are the initial choice for treating *P. aeruginosa* infections due to their effectiveness, extensive antibacterial range, and quick action [3]. The World Health Organization has classified CRPA as one of the three Critical Priority pathogens. Multidrug resistant *P. aeruginosa* (MDR-PA) infection, is associated with a 20% higher mortality rate, an excess cost of treatment, and increased length of stay and readmission rates compared to non- MDR *P. aeruginosa* [4].

More recently, the notion of difficult-to-treat resistance (DTR) has been proposed by *Kadri et al.* defining isolates that are non-susceptible to all first-line antipseudomonal agents (β-lactams, carbapenems, fluoroquinolones), thereby forcing the use of second-line or more toxic alternatives [5]. Infections due to MDR-PA which is defined as *P. aeruginosa* non-susceptible to at least one antimicrobial agent in three or more classes of antibiotics) [6], or difficult-to-treat resistance *Pseudomonas aeruginosa* (DTR-PA) are associated with various clinical and patient factors as historical antibiotic exposure (especially broad-spectrum agents), recent hospitalization or ICU stay, use of ventilators or vasopressors, tracheostomy, prior surgery, and prior colonization or infection [7].

The Antibacterial Resistance Leadership Group (ARLG) initiated in 2013 and located at Duke University North Carolina has done efforts to define the clinical and molecular patterns of CRPA. The agenda of this group focusses on 3 key areas: Gram-positive and Gram-negative infections, in addition to its diagnostics. Various resistant mechanisms are displayed by MDR-PA leading to the complexity of its resistance profile. Primarily, repression or inactivation of the carbapenem porin *OprD*. along with the overexpression of the chromosomal cephalosporinase *AmpC*, leads to reduced sensitivity to carbapenems [8]. Similarly, the increased function of efflux pump systems like *MexB* and *MexY* play a role in meropenem resistance [9].

Production of carbapenemases as a consequence of horizontal gene transfer through plasmids or integrons, has been increasingly reported over the last decade [10]. Carbapenemases includes *Klebsiella pneumoniae* carbapenemases (KPCs) and Guiana extended-spectrum (GES) enzymes, metallo β-lactamases (MβLs), in addition to oxacillinases (OXAs) are recognized for their effectiveness in hydrolyzing carbapenems. These mechanisms together or separately, enhance *P. aeruginosa* resistance to carbapenems [11].

Molecular methods give early knowledge of the type of carbapenemase which can improve antibiotic treatment selection and shorten the time needed for diagnosis.Moreover, these methods are helpful for epidemiological surveillance to determine the prevalence of carbapenemase genes in different settings [12]. We aimed to trace the emergence of DTR-PA isolates among the studied CRPA isolates and to identify their resistance mechanisms to carbapenems.

## Methods and materials

### Bacterial isolates

This study is a cohort study and represents a continuation of our previously published work [13] investigating CRPA isolates collected from hospitalized patients in Theodor Bilharz Research Institute between 2021 and 2023. Fifty-two (52) isolates were chosen from the previous collection of CRPA isolates and was used in the current study for extended phenotypic and molecular analyses. The 52 CRPA isolates were resistant to both imipenem, and meropenem as that was the most common resistance profile of CRPA from the clinical samples delivered to Microbiology Lab.

### Bacterial identification and antimicrobial susceptibility testing

Isolation, identification, and antimicrobial susceptibility testing were performed as previously described [14]. Briefly, isolates were identified using oxidase and catalase tests and species identification was done by VITEK2 compact system (BioMérieux, France). Susceptibility testing was conducted by the disk diffusion method in accordance with CLSI guidelines [14], and VITEK2 System according to manufacturer instructions. We aimed to investigate antibiotic sensitivity of the isolates to antibiotics that defines DTR-PA, that included (imipenem, meropenem, tazobactam/ piperacillin, ceftazidime, cefepime, aztreonam and ciprofloxacin) [5], in addition to aminoglycosides (amikacin and tobramycin) as second line therapy [12].

### DNA extraction and molecular detection of resistance genes

DNA extraction for 52 *P. aeruginosa* isolates resistant to both imipenem and meropenem was performed using the boiling method [15]. Conventional PCR was used to detect carbapenemase-encoding genes (*bla*_KPC_, *bla*_NDM_, *bla*_VIM_, *bla*_IMP_, *bla*_OXA−48_). PCR primers and conditions for previously investigated genes were described earlier [16, 17], while newly investigated targets were amplified using protocols detailed below.

### Detection of altered expression of *MexB*, *MexY *and *OprD *genes by Real Time -PCR (RT-PCR)

Repression of membrane porin *OprD* gene and overexpression of efflux pump *MexB* and *MexY* genes were detected by quantitative real time PCR (qRT-PCR). Extraction of RNA was performed using RNeasy Mini kit (QIAGEN, USA) as manufacturer’s instructions. Extraction was performed from the 52 CRPA isolates cultured on MacConkey’s agar after suspension in 4 ml Luria-Bertani (LB) broth medium (Thermo Fisher, USA) for 24 h at 37 °C.

First-strand cDNA was synthesized by reverse transcription by *QuantiTect*^*®*^ *Reverse Transcription* Kit (Qiagen, USA) that enables gene expression analysis by real-time two-step RT-PCR and the concentration of the resulting template was measured spectrophotometrically at OD260.

Qualified cDNA samples were stored at − 20 °C until further use. Quantitative real-time PCR was then carried out using a standard SYBR Green master mix (*2QuantiTect*^*®*^ SYBR^®^ *Green PCR*,* Qiagen*,* USA*) on a real-time PCR instrument *Quantstudio5 Real-Time PCR* (Thermo-Fisher Scientific, USA). The cDNA was diluted to a final concentration of 10 ng per reaction. The primers for RT-PCR (Table 1) were used as described before [18]. The thermal cycling conditions were as follows: initial denaturation at 95 °C for 15 min, followed by 40 cycles of 94 °C for 20s, 60 °C for 20s and 72 °C for 30s.

**Table 1:** Primers used in detection of *MexB*, *MexY* and *OprD* by Real Time PCR

Target gene	Nucleotide sequences (5’-3’)	Length in Bp	Reference
MexB	F; CAA GGG CGT CGG TGA CTT CCA G *R*; ACC TGG CAA CCG TCG GGA TTG A	272	[18]
MexY	F; GGA CCA CGC CGA AAC CGA ACG *R*; CGC CGC AAC TGA CCC GCT ACA	522	[18]
*OprD*	F; CGA CCT GCT GCT CCG CAA CTA R; TTG CAT CTC GCC CCA CTT CAG	242	[18]
*Rspl*	*Rspl-1;* GCTGCAAAACTGCCCGCAACG *Rspl-2;* ACCCGAGGTGTCCAGCGAACC	249	[18]

**Fig. 1:**
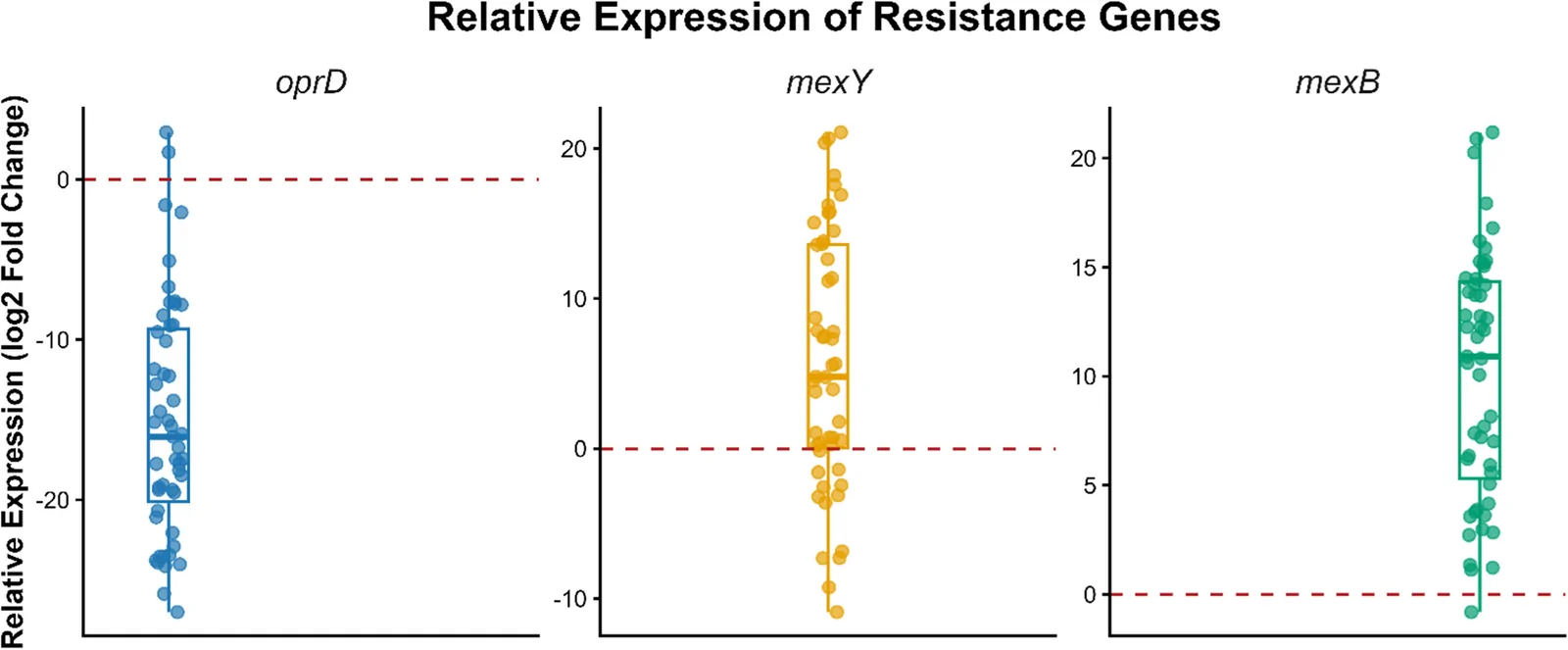
Relative expression of *oprD*, *mexY*, and *mexB* in carbapenem-resistant *Pseudomonas aeruginosa* clinical isolates. Relative gene expression was calculated using the comparative 2^-ΔΔCt method and presented as log fold change relative to the control isolate. Each point represents one clinical isolate, while boxplots indicate the median and interquartile range. The dashed horizontal line represents the reference value (log₂ fold change = 0)

**Fig. 2:**
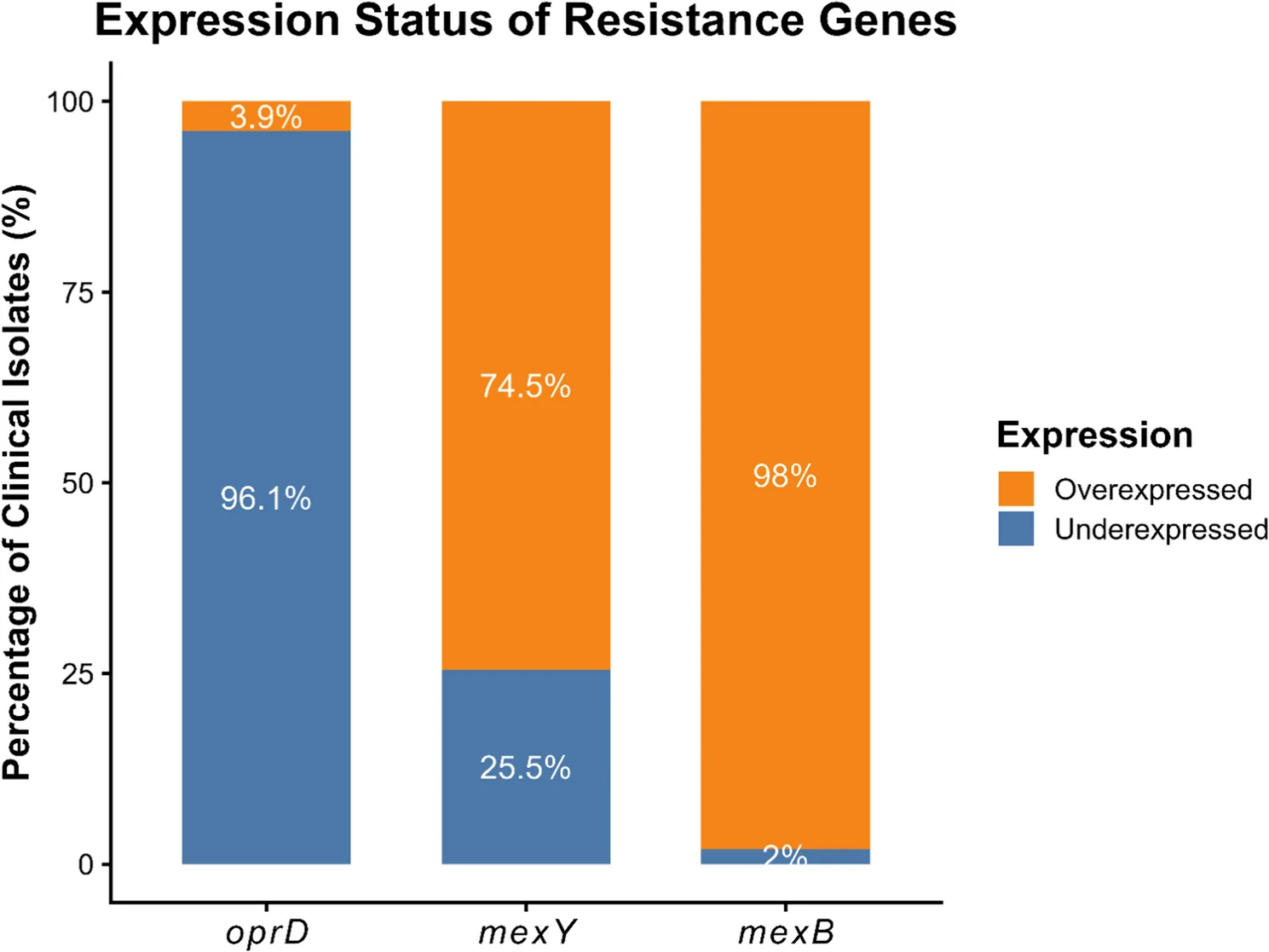
Expression status of resistance-associated genes among carbapenem-resistant *Pseudomonas aeruginosa* clinical isolates. Bars represent the percentage of isolates exhibiting down regulation or overexpression relative to the control isolate for each gene investigated

**Fig. 3:**
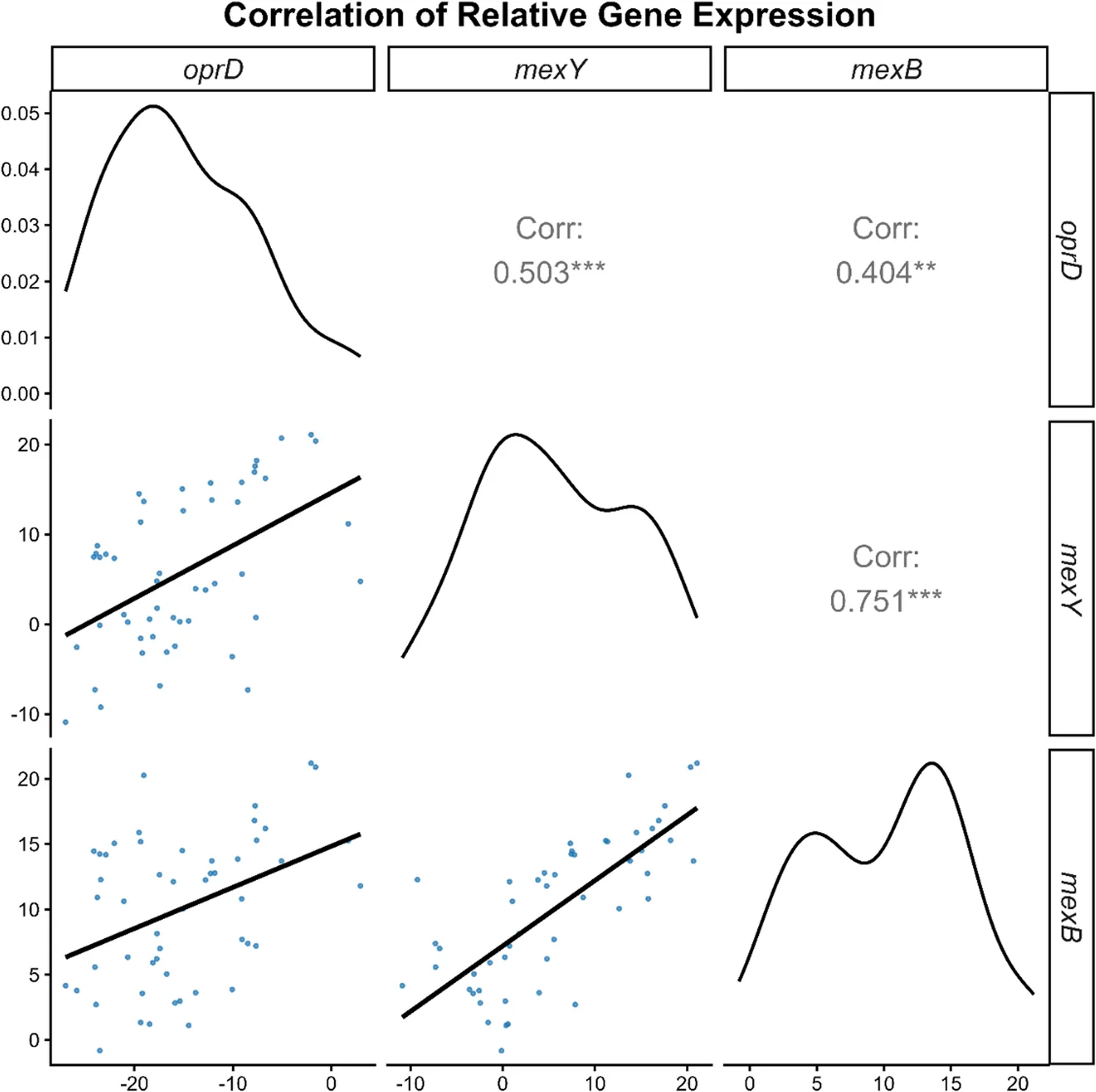
Correlation between the relative expression of *oprD*, *mexY*, and *mexB*. Pairwise scatter plots illustrate the relationships between log fold change values of the investigated genes. Pearson correlation coefficients are displayed in the upper panels, while regression lines are shown in the lower panels

A Standard curve analysis was performed at the end of each run. Gene Expression was normalized against the *rpsl* housekeeping gene of the same isolate. In addition, relative expression of *oprD*, *mexY*, and *mexB* in CRPA clinical isolates was performed. Data were reported as the relative expression of *MexB*, *MexY* genes and *OprD* compared to the same genes in the *P. aeruginosa* (ATCC 27853) reference isolate which was assigned a value of 1.0. (Figs. 1, 2 and 3).

Reduced *OprD* expression was considered significant when it was < 30% compared to that of the *P. aeruginosa* PAO1 reference isolate [19].

Melting Curve Analysis was done after the 40 cycles to assess the quality of the results; the presence of a single peak signified the occurrence of amplification in the target sequence alone and indicated that the data was safe for interpretation.

### Statistical analysis

Results were expressed as number (%). Comparison between parameters data was performed using Chi Square test. Agreement between different methods of detection was performed using kappa coefficient test. Diagnostic indices (sensitivity, specificity, positive, negative and efficacy) were performed according to Statistical Package for Social Sciences (SPSS) version 21. Predictive (P) values < 0.05 were considered significant and < 0.01 was considered highly significant.

## Results

### Antimicrobial susceptibility profile

According to the results by VITEK2, the highest resistance rate was identified among ciprofloxacin (96%), followed by piperacillin (94%). Tazobactam showed a resistance rate of 85%. Among aminoglycosides tobramycin showed resistance of 85%, followed by amikacin 79%. Ceftazidime showed a resistance of (83%), cefepime (81%) and aztreonam was the most sensitive antimicrobial being resistant in only (35%) of isolates.

Carbapenem resistant *P. aeruginosa* isolates were classified by resistance phenotypes into: multidrug-resistant *P. aeruginosa* (MDR-PA) that includes isolates non-susceptible to at least one agent in three or more antimicrobial classes; extensively drug-resistant *P. aeruginosa* (XDR-PA) including isolates that are non-susceptible to all but two or fewer antimicrobial classes; difficult-to-treat resistance (DTR) for isolates non-susceptible to all first-line anti-pseudomonal agents, including ceftazidime, cefepime, aztreonam, meropenem, imipenem and ciprofloxacin. Finally, carbapenem-resistant *P. aeruginosa* (CRPA) – resistant to carbapenems but did not fit into any of the previous categories [6, 5].

Applying the DTR definition resulted in three main resistance phenotypes for CRPA: DTR-PA representing 46% (20/52), where all isolates in this category were initially considered XDR, however being resistant to all first line antipseudomonal antibiotics made them fit to DTR definition, XDR-PA (non-DTR isolates) representing 38% (20/52) of all CRPA. Four isolates (8%; 4/52) were categorized as MDR-PA, and four CRPA isolates did not fit into any definition so they remained non-categorized Table 2. Where aztreonam showed significantly higher susceptibility in XDR-PA than DTR-PA and MDR-PA, while MDR-PA also exhibited significantly greater susceptibility (*P* < 0.001).

**Table 2:** Susceptibility profiles of different antibiotics in relation to resistance phenotypes

Resistance Phenotype*	Sensitivity to antibiotics % (*n*)					
	Ceftazidime (CAZ)	Cefepime (FEP)	Tazobactam/ piperacillin (TZP)	Aztreonam (ATM)**	Ciprofloxacin (CIP)	Aminoglycosides (AK/TOB)^c^
DTR-PA (*n* = 24)	0	0	0	0	0	8 (2)
XDR-PA (*n* = 20)	0	0	5 (1)	95 (19)	0	5 (1)
MDR-PA (*n* = 4)	10 (4)	100 (4)	75 (1)	75 (1)	0	10 (4) to AK 0 to TOB
CRPA (= 4)	100 (4)	100 (4)	100 (4)	0	50 (*n* = 2)	100 (4)

*XDR-PA Extensively drug resistant *P. aeruginosa*, *DTR-PA* Difficult-to-treat *P. aeruginosa*, *MDR-PA* Multidrug resistant *P. aeruginosa*, *CRPA* Carbapenem resistant *P. aeruginosa*

** Aztreonam sensitivity differed significantly across resistance phenotypes (*P* < 0.001)

^a^Isolate sensitive to Tazobactam/ piperacillin was positive to porin loss and over expression of efflux pump

^b^All isolates sensitive to aztreonam were positive for MβLs (NDM (*n* = 18), VIM (*n* = 1)

^C^*AK* Amikacin, *TOB* Tobramycin

^d^One isolate was sensitive to both Amikacin (AK) and Tobramycin (TOB), the other 2 isolates showed sensitivity to only one agent of them

### Carbapenemases genes

Detection of carbapenemase genes by conventional PCR revealed that 81% (42/ 52) isolates had at least one of the carbapenemases genes (*bla*_KPC_, *bla*_NDM,_ *bla*_VIM_, and *bla*_IMP_, and *bla*_OXA−48_). Alternatively, 19% (10/52) isolates tested negative for all the genes included in the study suggesting that these isolates exhibit another resistance mechanism.

Genes encoding MβLs (including NDM and VIM enzymes) were predominant in DTR-PA and XDR-PA isolates representing 92% and 95% respectively. The *bla*_NDM_ was the predominant gene in DTR-PA and XDR-PA found in 75% (18/24) and 90% (18/20) respectively, followed by *bla*_VIM_ 54% (13/24) and 45% (9/20) respectively, whereas *bla*_OXA−48_ was found in DTR-PA in 38% (9/24) and they were always in combination with other genes of MβLs (5 isolates with *bla*_NDM_,1 isolate with *bla*_VIM_, and 3 isolates with both MβLs genes). In XDR-PA *bla*_OXA−48_ was found in 30% (6/20) of the isolates and was also present in combination with MβLs (2 with *bla*_NDM_ and 2 with *bla*_VIM_). Among isolates categorized as MDR-PA, only one isolate was found positive to *bla*_VIM_ (25%; 1/4). No carbapenemases encoding genes were detected in CRPA with non-categorized resistance phenotype. Two carbapenemase genes (*bla*_KPC_ and *bla*_IMP_) weren’t detected in any of our isolates (Table 3). None of the tested carbapenemase genes showed a statistically significant difference between the different resistance phenotypes included.

**Table 3:** Type of carbapenemases detected in different resistance phenotypes

Resistance Phenotype	Carbapenemases				
	bla_KPC_%	MβLs (%)			bla_OXA−48_%
		bla_NDM_ %	bla_VIM_ %	bla_IMP_ %	
DTR-PA (*n* = 24)	0	75	54	0	38
XDR-PA (*n* = 20)	0	90	45	0	30
MDR-PA (*n* = 4)	0	0	25	0	0
CRPA (*n* = 4)	0	0	0	0	0

*XDR-PA* Extensively drug resistant *P. aeruginosa*, *DTR-PA* Difficult-to-treat *P. aeruginosa*, *MDR-PA* Multidrug resistant *P. aeruginosa*, *CRPA* Carbapenem resistant *P. aeruginosa*

### *MexB*, *MexY* and *OprD* genes

Of the XDR-PA isolates, 95% (19/20) showed overexpression of the efflux pump genes *MexB* and *MexY*, as well as downregulation of *OprD*, which resulted in the loss of outer membrane porin. Furthermore, all DTR-PA and MDR-PA isolates (100%) had overexpressed efflux pump genes (Table 4, Figs. 1, 2 and 3).

**Table 4:** Mechanisms of resistance to carbapenems in relation to different resistance phenotypes

Resistance Phenotype	Carbapenemase production + Porin loss+ Overexpressed efflux pump (*n*%)	Porin loss + Overexpressed efflux pump (*n*%)	Carbapenemase production + Porin loss (*n*%)	Carbapenemase production + Overexpressed efflux pump (*n*%)	Overexpressed efflux pump (*n*%)
DTR-PA (*n* = 24)	22 (92)	2 (8)	0	0	0
XDR-PA (*n* = 20)	17 (85)	1 (5)	1 (5)	1 (5)	0
MDR-PA (*n* = 4)	1 (25)	2 (50)	0	0	1 (25)
CRPA (= 4)	0	2 (50)	0	0	2 (50)

*XDR-PA* Extensively drug resistant *P. aeruginosa*, *DTR-PA* Difficult-to-treat *P. aeruginosa*, *MDR-PA* Multidrug resistant *P. aeruginosa*, *CRPA* Carbapenem resistant *P. aeruginosa*

*OprD* was strongly down regulated across almost all isolates with most values between about − 10 and − 25. MexY and MexB were mostly upregulated between 0 and 15 and 0–20 respectively (Fig. 1).

Porin loss and efflux pump resistance mechanisms were detected in 8% (2/24) of DTR-PA isolates, 5% (1/20) of XDR-PA isolates, 50% (2/4) of MDR-PA isolates and 50% (2/4) of CRPA isolates. Efflux pump resistance mechanism was detected in only 25% (1/4) of MDR-PA isolates and in 50% (2/4) of CRPA isolates. Among all 52 studied isolates, three 6% (3/52) exhibited the presence of efflux pump resistance mechanism, and 2% (1/52) showed presence of carbapenemases production with efflux pump and another one isolate showed carbapenemases production with porin resistance mechanisms (Table 4).

Loss or down regulation of *OprD* porin was identified in 100% of DTR-PA, 95% of XDR-PA and 83% of MDR-PA. Similarly, overexpression of the efflux pump was detected in 100% of DTR-PA as well as in all MDR-PA and CRPA isolates. Co-existence of resistance mechanisms in relation to their resistance phenotype was shown in Table 4.

The prevalence of isolates harboring the maximum resistance mechanisms of carbapenemase production, porin loss, and overexpressed efflux pumps was higher in DTR-PA than in XDR-PA isolates (92% vs. 85.0%), although this difference was not statistically significant (*P* = 0.561). Comparisons involving MDR-PA allowed significance difference between this group and other groups but small sample size of this group limits the reliability of the test.

## Discussion

The emergence of DTR-PA represents a significant clinical challenge, as it has been associated with poor prognosis and mortality risk compared to those with CRPA [20]. Because carbapenem resistance in *P. aeruginosa* arises from the interaction of several resistance mechanisms rather than a single determinant, it is especially difficult to treat. Non-enzymatic mechanisms, such as loss or decreased expression of the *OprD* porin, overexpression of multidrug efflux pumps, AmpC over production, and their combined effects, are crucial in mediating resistance and frequently coexist within the same isolate, in addition to the acquisition of carbapenemase genes [21].

Several classifications for antibiotic resistance were proposed where Magiorakos et al. being the most widely used. This classification includes (MDR), (XDR), and pandrug-resistant (PDR), based on the number of antibiotic classes showing activity in vitro [6], thus weighing all antibiotics equally regardless of their actual effectiveness [22]. The (DTR-PA) classification was first proposed by *Kadri et al.*., who defined it as non-susceptibility to all tested β-lactams (including carbapenems), fluoroquinolones, antipseudomonal penicillins (piperacillin-tazobactam), cephalosporins (ceftazidime and cefepime), aztreonam and carbapenems (imipenem and meropenem) [5]. This is based on the concept that nonsusceptibility to all first-line agents usually leads to use of second-line agents (such as aminoglycosides, or polymyxins) which are characterized by poorer pharmacokinetic properties and increased risk of toxicity, thus resulting in unfavourable outcome [23]. So, the application of the DTR definition among surveillance studies has been advised for better comparison of clinically relevant resistance mechanisms among *P. aeruginosa* in various geographic locations [24].

Although the emergence of DTR-PA represents a significant clinical challenge compared to those with CRPA [20], studies addressing DTR-PA in Egypt are very scarce. Therefore, we aimed to characterize DTR-PA isolates among our CRPA; regarding their resistance mechanisms to carbapenems and their antimicrobial susceptibility profiles.

All our CRPA isolates were resistant to both carbapenems; imipenem and meropenem, although that may represent high resistance to carbapenems, however those were the available isolates for the current study, and they actually represented the predominant phenotype of carbapenem resistance in our hospital. All DTR-PA isolates were subsets of XDRs, where XDR-PA (not fitting into the DTR definition) showed sensitivity to any of the first line antipseudomonal antimicrobial agents; mainly aztreonam (*n* = 19) and piperacillin/tazobactam (*n* = 1). Thus, the existing resistance phenotypes were: DTR-PA, XDR-PA, MDR-PA and CRPA representing 46%, 38%, 8% and 8% respectively. Four CRPA did not fit into any category. Same categorization was applied previously by *Shabi et al.* [24].

We did not use the term of pan-drug resistant (PDR), as we did not include other antibiotics as colistin that could be used as a last option of treatment regardless of its significant risks.

This alarming high rate of DTR-PA among carbapenem resistant isolates in this study represents a serious therapeutic challenge in health care facilities and surpasses most regional and global findings. However, high rate (82.4%) have been found in a former Egyptian study by *Fayed and colleagues* [25], although noticeably lower rates were detected in Lebanon 15.3%, reflecting geographical discrepancy [26]. In addition, DTR-PA rates also show considerable variation across the Arabian Gulf countries 12.5% in Saudi Arabia, 19% in Kuwait, and 25.9% in Qatar [27]. higher prevalence of DTR reported in our study may reflect a growing trend of resistance in the Egyptian healthcare settings, which could be attributed to variations in infection control practices and antimicrobial programs across different regions.

Most of the antibiotics were ineffective against the studied isolates. Ciprofloxacin showed the highest resistance rate, followed by piperacillin, similar results were obtained in former studies in Egypt [28]. High rate of resistance to ciprofloxacin was also obtained in China [29]. Among aminoglycosides, isolates showed higher resistance to tobramycin than amikacin. Comparable results were obtained by *Fayed* and colleagues [25] in Egypt and by *El Badawi* and his team [30] from Sudan. On the contrary, resistance to aminoglycosides was low in Kingdom of Saudi Arabia [31]and in China [32]. This may be due to selective pressure applied as a result of exposure to different antimicrobial agents used under varying hospital antimicrobial stewardship programs.

Applying the DTR-PA categorization highlighted the CRPA with the least susceptibility profiles compared to other resistance phenotypes (Table 2), thus marked the isolates that most likely to present significant clinical challenges. The sensitivity to aztreonam in almost all XDR-PA isolates was remarkable 95% (19/20), whereas resistance to aztreonam nearly defined all DTR-PA isolates. This sensitivity profile points to the endemicity of MβLs (especially NDM enzymes), as all the isolates sensitive to aztreonam within the XDR-PA (non-DTR) phenotype group were found positive to either *bla*_NDM_ or *bla*_VIM_ or both (Table 3). High rates of NDM carbapenemases correlates with the endemicity reported in Middle Eastern and developing countries [33, 28], thus confirming the critical situation escalated by these enzymes in endemic regions. In non-categorized CRPA isolates, tazobactam/piperacillin maintains 100% sensitivity, thus emphasizing how understanding resistance phenotype is crucial prior to treatment selection Table 2. Previous studies in Egypt had reported that *bla*_NDM_ was the most predominant carbapenemase gene [34, 35]. On the other hand, another study performed in Minia University reported that *bla*_VIM_ (57.9%) was the most common carbapenemase followed by *bla*_OXA−48_ (50.8%) and the least common gene was *bla*_NDM_ (3.5%) [11]. Former studies supported our finding which denoted that *bla*_IMP_ has a little role in carbapenem resistance in *P. aeruginosa* in Egypt [36, 37]. The rise of MβLs could greatly limit available and newly developed therapeutic options [35].

Reports identifying *bla*_OXA−48_ producing *P. aeruginosa* in Egypt has been recently identified [11, 38]. Carbapenem-resistant *bla*_OXA−48_ positive *P. aeruginosa* was also reported in Iran, Sudan, and India [39–41]. Although OXA-type carbapenemases are rarely identified in *P. aeruginosa*. It has been suggested that carbapenem resistance genes were transmitted to *P. aeruginosa* via mobile genetic elements from other microorganisms such as Enterobacterales and *A. baumannii* colonizing in burn units that implement poor infection control practical measures [11]. However, in our study *bla*_OXA−48_ was always detected in combination with other genes encoding MβLs (as *bla*_NDM_ or *bla*_VIM_ or both), thus reflecting the minor role played by these realatively weak carbapenemases in CRPA when compared to MβLs.

The current study revealed that *MexB* and *MexY* genes contribute as a major factor in antibiotic resistance mechanisms. The detection of over expression of *MexB* gene in most of our isolates demonstrated its contribution to multidrug resistance and the constitutive expression of *MexAB-OprM* efflux pump in *P. aeruginosa*, as an intrinsic mechanism of resistance to multiple classes of antibiotics, including β-lactams, and fluoroquinolones. However, an Egyptian study conducted by *Elbrolosy et al.* [36]. have noted the occasional down regulation or mutation of the *mexB* gene. On the other hand, *MexY* efflux pump was the most prominent resistance mechanism in other reports [42, 43, 29]. The predominance of *MexB* over *MexY* in our study may reflect differences in isolate populations, local antibiotic selection pressure, geographic epidemiology, and the resistance phenotype of the investigated DTR-PA isolates [44]. 

Generally, reduced expression of *OprD* confers resistance to imipenem but not usually to meropenem, depending on the type of *OprD* gene mutations [45]. Down regulation of *OprD* in the current study aligned with a study reported by *Ghoneim et al.* [46]. Our findings are consistent with previous reports from Greece, indicating that carbapenem resistance in *P. aeruginosa* is predominantly mediated by the loss or reduced expression of the outer membrane porin *OprD*, often combined with overexpression of the *MexAB*-*OprM* efflux pump. Moreover, *OprD* deficiency commonly occurs in combination with overexpression of the *MexAB-OprM* efflux system, producing a synergistic reduction in intracellular antibiotic accumulation and leading to higher levels of carbapenem resistance even in the absence of carbapenemase production. These observations support the concept that non-enzymatic mechanisms, particularly *OprD* loss coupled with efflux pump overexpression, remain major drivers of carbapenem resistance in *P. aeruginosa*, especially among difficult-to-treat clinical isolates [47].

Thus efflux pump activity and carbapenemase production played significant roles in CRPA [48].

Regarding the mechanisms of carbapenem resistance within each resistance phenotype, all the study isolates harbored multiple mechanisms of resistance to carbapenems except two isolates (50%) of CRPA non-categorized group. The accumulation of multiple resistance mechanisms—including carbapenemase production, porin loss, and efflux pump overexpression—was most frequently observed among DTR-*P. aeruginosa* isolates (92%), followed by XDR-*P. aeruginosa* isolates (85%). In contrast, only 25% of MDR-*P. aeruginosa* isolates exhibited the simultaneous presence of these resistance mechanisms [36]. On the other hand, porin loss and overexpressed efflux pumps (with undetected carbapenemases) marked the uncategorized CRPA isolates thus indicating impaired outer membrane permeability as major factors in non-carbapenemase producers’ resistance to carbapenems (Table 4).

Our results are in line with a recent national investigation by *Ramadan et al.* which found that isolates that did not produce carbapenemase had a very high rate of reduced expression of *oprD* (89.5%), while, *MexB* overexpression was the most common among the efflux pumps (76.3%) and MexY was over expressed in (23.7%) [49]. In the meantime, a study from Dubai in the Gulf Region found that decreased expression of Outer membrane impermeability *OprD* was observed in 73% of CRPA isolates and 75.6% displayed overproduction of MexAB-OprM [50]. In a study in Japan, 69% of MDR-PA isolates showed downregulation of *OprD* while efflux pump genes were over expressed in 67% of MDR-PA isolates [51]. 

Our study has limitations as all CRPA in the study were resistant to both carbapenems, however this phenotype comprises the majority of all CRPA in the hospital. In addition, we did not include colistin susceptibilityas broth microdilution was not available as a reference method, however according to CLSI M100 guidelines, only intermediate or resistant breakpoints are defined for PA. Also, all the isolates were from the same hospital, further multi-center studies with larger sample sizes is required, and whole genome sequencing (WGS) is recommended to further understand the characterization and the epidemiology of these isolates.

## Conclusion

It is essential to continuously screen resistance determinants to allow rapid diagnosis and targeted treatment. In this study, the prevalent resistance mechanisms in DTR-PA were the coexistence of *OprD* downregulation, overexpression of *MexB* and *MexY*, in addition to carbapenemase production. Applying the DTR-PA definition highlighted certain subsets of highly resistant CRPA that most likely to cause challenging clinical situations. Predominance of *bla*_NDM_ gene, confers a high degree of resistance to currently available antibiotics, in addition to the spread of antimicrobial resistance among bacterial populations. Further studies with larger sample size from multiple healthcare centers are needed to understand the mechanisms of antibiotic resistance and the outcomes of infections caused by DTR-PA across different infection sites and clinical scenarios.

## Data Availability

The datasets supporting the conclusions of this article are included within the article.

## Abbreviations

CRPA: Carbapenem resistant *Pseudomonas aeruginosa*
MDR: Multi-drug resistant
*P. aeruginosa*: *Pseudomonas aeruginosa*
MDR *P. aeruginosa*: Multidrug resistant *P. aeruginosa*
KPCs: *Klebsiella pneumoniae* carbapenemases
GES enzymes: Guiana extended-spectrum enzymes
MβL: Metallo β-lactamases
OXAs: Oxacillinases
DTR: Difficult to treat
TBRI: Theodor Bilharz Research Institute
AST: Antimicrobial Susceptibility Testing
IPM: Imipenem
MEM: Meropenem
TIC: Ticarcillin
T/C: Icarcillin/clavulanic
TZP: Piperacillin/tazobactam
FEP: Cefepime
AK: Amikacin
CN: Gentamycin
TOB: Tobramycin
CIP: Ciprofloxacin
ATM: Aztreonam
*CLSI*: Clinical and Laboratory Standards Institute
MIC: Minimum inhibitory concentration
PCR: Polymerase chain reaction
RT-PCR: Real Time -PCR
qRT-PCR: Quantitative real time PCR
XDR: Extensively drug-resistant
